# Cardiac glycosides suppress the maintenance of stemness and malignancy via inhibiting HIF-1α in human glioma stem cells

**DOI:** 10.18632/oncotarget.16714

**Published:** 2017-03-30

**Authors:** Dae-Hee Lee, Sang Cheul Oh, Amber J. Giles, Jinkyu Jung, Mark R. Gilbert, Deric M. Park

**Affiliations:** ^1^ Division of Oncology/Hematology, Department of Internal Medicine, Korea University College of Medicine, Seoul, Republic of Korea; ^2^ Neuro-Oncology Branch, CCR, NCI, National Institutes of Health, Bethesda, MD, USA

**Keywords:** cancer stem cell, digitoxin, cardiac glycoside, hypoxia, HIF

## Abstract

Tissue hypoxia contributes to solid tumor pathogenesis by activating a series of adaptive programs. We previously showed that hypoxia promotes the preferential expansion and maintenance of CD133 positive human glioma stem cells (GSC) in a hypoxia inducible factor 1 alpha (HIF-1α)-dependent mechanism. Here, we examined the activity of digitoxin (DT), a cardiac glycoside and a putative inhibitor of HIF-1α, on human GSC *in vitro* and *in vivo*. During hypoxic conditions (1% O_2_), we observed the effect of DT on the intracellular level of HIF-1α and the extracellular level of vascular endothelial growth factor (VEGF) in human GSC. We found that DT at clinically achievable concentrations, suppressed HIF-1α accumulation during hypoxic conditions in human GSC and established glioma cell lines. DT treatment also significantly attenuated hypoxia-induced expression of VEGF, a downstream target of HIF-1α. Exposure to DT also reduced hypoxia-induced activation of the extracellular signal-regulated kinase 1/2 (ERK1/2) signaling pathway. Furthermore, DT potently inhibited neurosphere formation, and decreased CD133 expression even at concentrations that were not overtly cytotoxic. Lastly, treatment with DT reduced GSC engraftment in an *in vivo* xenograft model of glioblastoma. Intraperitoneal injections of DT significantly inhibited the growth of established glioblastoma xenografts, and suppressed expression of HIF-1α and carbonic anhydrase (CA9), a surrogate marker of hypoxia. Taken together, these results suggest that DT at clinically achievable concentration functions as an inhibitor of HIF-1α, worthy of further investigations in the therapy of glioblastoma.

## INTRODUCTION

Therapeutic targeting of the cancer stem cell subpopulation has potential for more durable treatment of cancer. However to date, relatively few specific targets have been identified. HIF was recently shown to regulate the tumorigenic capacity of glioma stem cells (GSC) in hypoxic conditions [1–3]. Glioblastoma (GB) is one of the most aggressive human cancers, and prognosis remains dismal. GB is characterized by rapid disease relapse even with standard therapy consisting of surgery, radiation therapy, and chemotherapy with median survival of 14 months [4]. A suggested reason for the highly refractory nature of GB is the presence of GSC, a subpopulation of tumor cells regarded as the source of tumor initiation and relapse. GSC may represent the tumor-initiating cells of GB, and are believed to contribute to the resistance to conventional therapies. Development of novel chemotherapeutic agents and treatment approaches against GB, particularly those that specifically target GSC, are thus likely to result in more effective and durable response. GSC are enriched in the perivascular niche and areas near necrosis [5], which in turn are associated with reduced oxygen tension, or hypoxia. Although hypoxia is often an inevitable outcome of the rapidly growing tumor outgrowing its lagging vascular supply, it confers certain advantages for tumor cells [6]. To maintain GSC growth in hypoxia, both HIF-1α and HIF-2α are required [7–9]. We previously demonstrated that hypoxia promotes maintenance and preferential expansion of GSC in a HIF-1α dependent mechanism [1]. Hypoxia also appears to play a key role in the maintenance of pluripotency of embryonic stem cells [10].

Digitalis (extracts from *Digitalis purpurea* and *Digitalis lanata*, specifically DT and digoxin) has been used as a cardiac drug for over 200 years. DT, a clinically approved cardiac glycoside for heart failure, has shown anti-cancer effects in many types of cancers [11]. For example, breast cancer patients receiving digitalis had improved outcomes compared to untreated patients [12]. Proposed mechanisms of action include inhibition of Na^+^/K^+^ ATPase pump, inhibition of topoisomerases, inhibition of angiogenesis, and interaction with the MAPK signaling cascade [11, 13, 14]. In our previous effort to screen repurposed small molecule drugs for cancer therapy, cardiac glycosides were unexpectedly among the top hits [15]. Intriguingly, an unbiased library screening conducted to search for HIF-1α inhibitors identified 20 compounds with 11 of these being cardiac glycosides [16].

Our previous work and that of others have demonstrated the importance of hypoxic signaling to the maintenance of GSC stemness as well as its proliferative capacity. In this study, we examined two cardiac glycosides used in the clinic, digoxin and DT, in GSC propagated in hypoxia as well as in xenograft models. We observed that only DT in clinically achievable concentration, was capable of suppressing HIF-1α and its downstream signaling in hypoxic conditions, leading to loss of GSC self-renewal capacity. These findings were further confirmed in orthotopic xenograft models of GB.

## RESULTS

### DT inhibits accumulation of HIF-1α in GSC exposed to hypoxia

**Figure 1:**
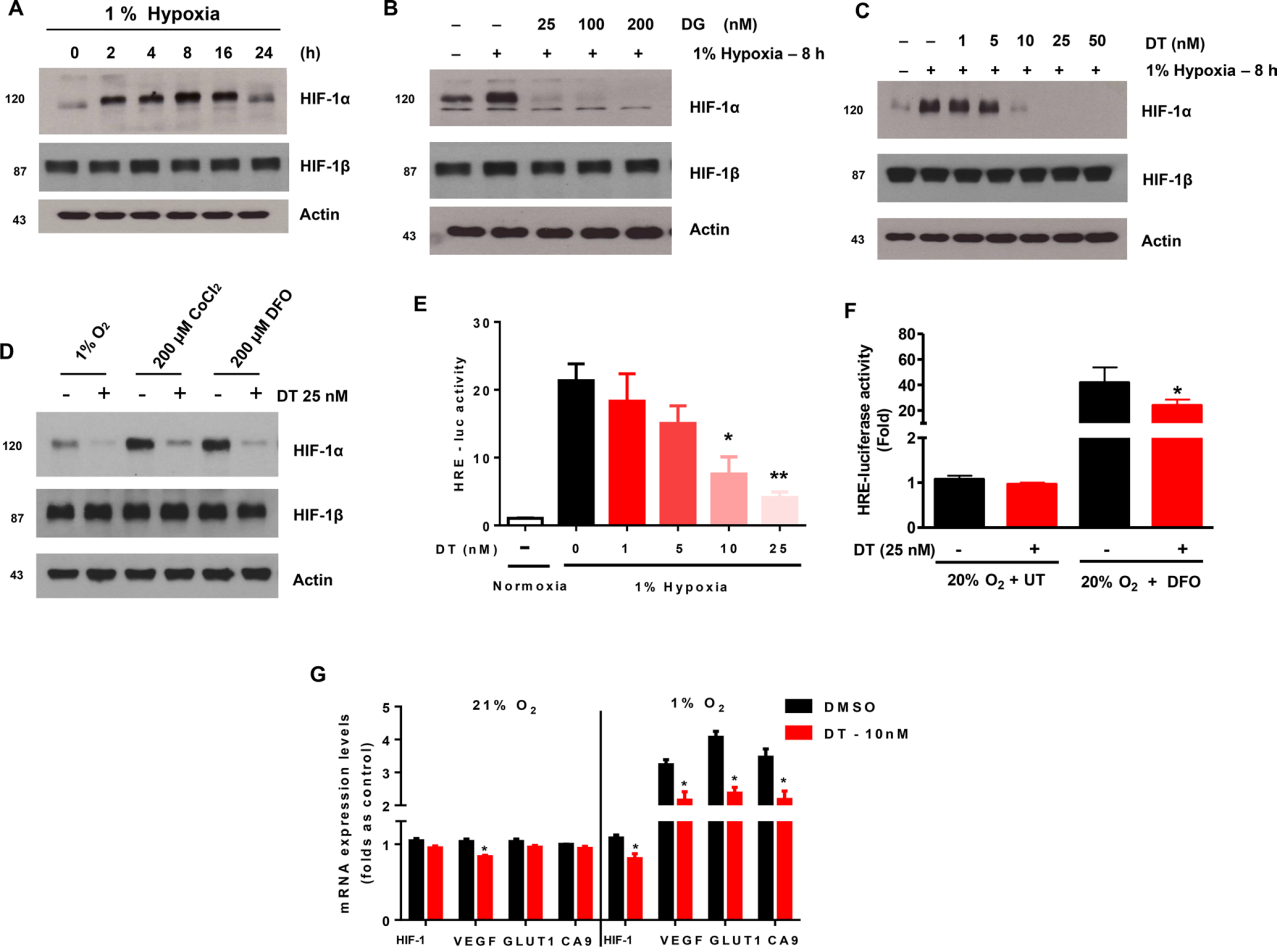
Cardiac glycosides inhibit the accumulation of HIF-1a in X01 GSC during hypoxia Cells were exposed to 1% O_2_ and treated with various concentrations of DG or DT for 8 h. (**A**) Kinetics of HIF-1a accumulation in hypoxic conditions. (**B**) Kinetics of HIF-1a accumulation during treatment with digoxin (DG) in hypoxic conditions. (**C**) Kinetics of HIF-1a accumulation during treatment with DT in hypoxic conditions. (**D**) Cells were exposed to 1% O_2_ hypoxia, CoCl2, and DFO in combination with DT for 8 h then harvested. (**E**) HRE-luciferase activity, after transfection with promoter constructs and treatment with DT, in GSCs under conditions of normoxia and hypoxia for 16 h. (**F**) HRE-luciferase activity of GSC treated with vehicle or DT under normoxia (20% oxygen + UT) or hypoxia-mimicking (20% oxygen + DFO) conditions. (**G**) The quantitative analysis of hypoxia-responsive gene expression under normoxia and hypoxia with DT for 16 h, evaluated as sample threshold value divided by HTERT value (bars, ± SD; *P < 0.05, DT compared to DMSO groups). Cell lysates containing equal amounts of protein (20 μg) were separated by SDS-PAGE and immunoblotted with anti-HIF-1α and anti-HIF-1β antibodies. Actin was used as a loading control.

We first examined if digoxin or DT could induce cytotoxicity, and if digoxin or DT-induced cytotoxicity was responsible for the observed suppression of HIF-1α protein level. The effects of cardiac glycosides on cell viability and drug-induced PARP-1 cleavage, were determined by trypan blue exclusion and Western blotting, respectively (Supplementary Figure 1A–1C). These graphs show dose dependent 48 hour survival of GSC exposed to digoxin and DT. However when treated for a shorter duration of 8 hours in hypoxia, clinically achievable concentrations of DT (10–25 nM) had no significant effect in viability (Supplementary Figure 1D). GSC incubated in 1% oxygen condition led to accumulation of HIF-1a within 2 hours and maintained protein expression in hypoxia (Figure 1A). Treatment of GSC propagated in hypoxia, with digoxin (DG) and DT led to reduction of HIF-1α protein expression (Figure 1B and 1C). However, while DT was capable of inhibiting HIF-1α expression at clinically achievable concentrations (10–25 nM), digoxin required higher concentration than used for cardiac therapy (2–3 nM). DT was capable of inhibiting HIF-1a accumulation induced by hypoxia-mimic chemicals Cobalt chloride (CoCl_2_) and Deferoxamine Mesylate (DFO), without cytotoxic activity (Figure 1D and Supplementary Figure 1D). To examine if these results represent a general mechanism, we extended these studies to four different cell lines including GSC (XO2 and XO3) and human glioma cells (U373 and T98G). HIF-1a accumulation was suppressed by treatment with DT in hypoxic conditions in all 4 additional cell lines (Supplementary Figure 4).

HIF-1 is an important transcription factor that binds to a number of genes via HIF responsive elements (HREs) to activate transcription [17–19]. Using cells transfected with an HRE-luciferase reporter construct, we found that DT significantly attenuated induction of luciferase activity in a dose-dependent manner (Figure 1E). DT displayed a similar inhibitory effect on DFO-induced promoter activity (Figure 1F). We next examined the effect of DT treatment on production of HRE-responsive genes (HIF-1α, VEGF, Glut1, and CA9). As shown in Figure 1G, expression of these genes was inhibited by DT during hypoxia. These results suggest that clinically relevant concentrations of DT can decrease hypoxia-induced HIF-1α protein accumulation and its downstream signaling pathways.

### DT inhibits hypoxia-induced activation of extracellular signal-regulated kinase1/2 in GSC

**Figure 2:**
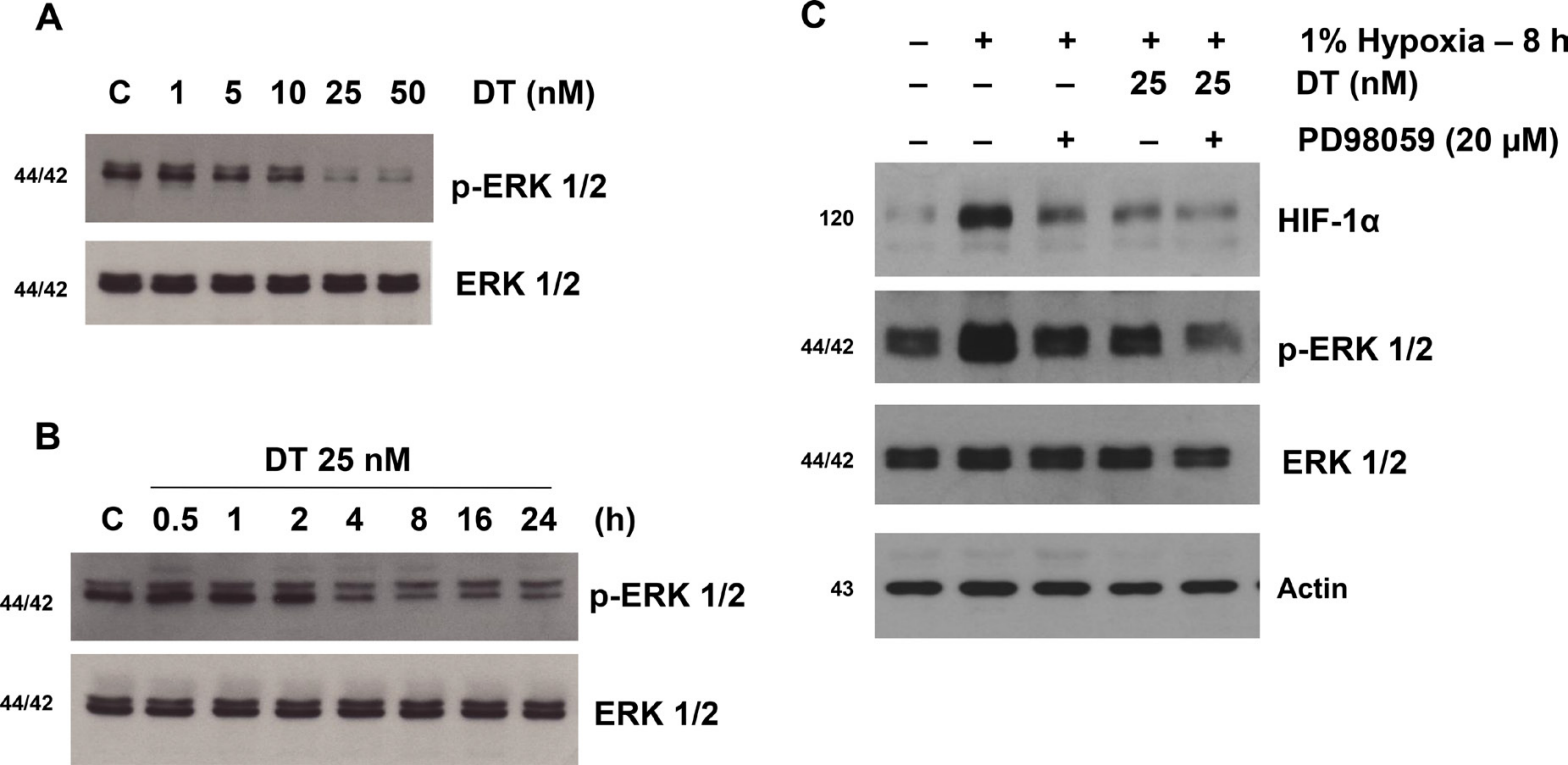
DT inhibits hypoxia-induced activation of extracellular signal-regulated kinase 1/2 (ERK1/2) in GSC Western blot analyses of X01 GSC cultured in 1% oxygen were performed. (**A**) p-ERK1/2 and total ERK1/2 of cells treated with vehicle or increasing concentrations of DT for 8 hours is shown. (**B**) Immunoblot shows p-ERK1/2 from cells cultured with vehicle or 25 nM DT at the indicated times. (**C**) Cells were treated with PD98509 (an ERK inhibitor) or vehicle for 30 min followed by treatment with DT or vehicle for 8 h. Cell lysates, containing equal amounts of protein (20 mg), were separated by SDS-PAGE and immunoblotted with anti-HIF-1α, anti-phospho-ERK (Thr202/Tyr204), or anti-ERK antibodies. Actin was used as a loading control.

Our previous results and that of others have demonstrated presence of crosstalk between HIF-1α and growth factor signaling cascades [1, 20–22]. Hypoxia by promoting HIF-1α stability can activate ERK1/2 signaling (Figure 2A). We explored if DT by inhibiting HIF-1α is capable of abrogating hypoxia-induced ERK1/2 activation. We found that DT treatment abrogated hypoxia-induced phosphorylation of ERK1/2 in a fashion comparable to direct inhibition of ERK1/2 (Figure 2B and 2C). Interestingly ERK inhibition led to reduction of HIF-1α level further suggesting presence of crosstalk between hypoxic and growth factor signaling cascades.

### DT inhibits hypoxic HIF-1α accumulation by inhibiting protein synthesis

**Figure 3:**
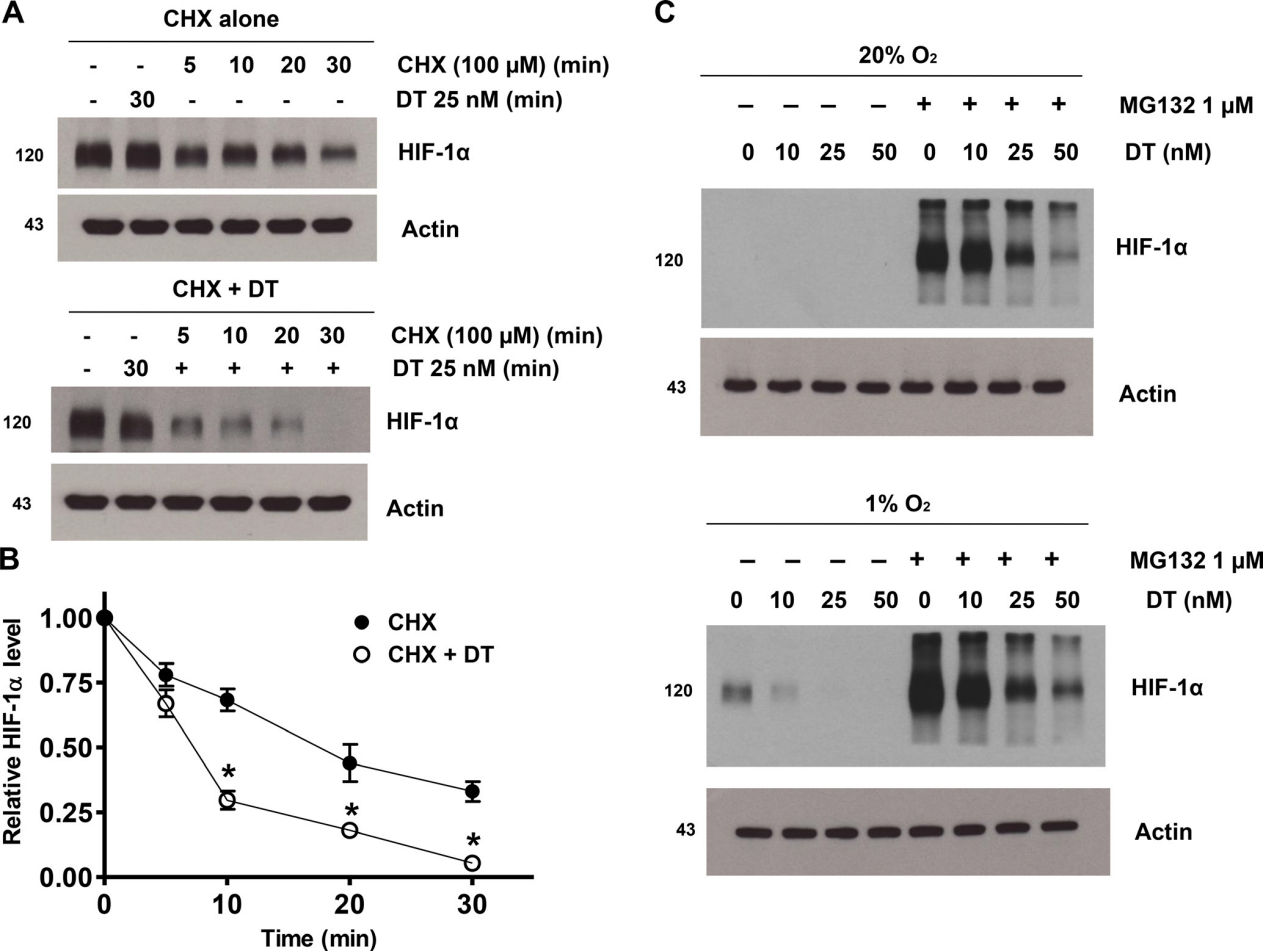
DT inhibits hypoxic HIF-1α accumulation by inhibiting protein synthesis (**A**) GSC were treated with solvent alone (CHX; top panel) or 25 nM DT (CHX + DT; bottom panel) for 1 h, followed by incubation with 100 μM CHX from 0 to 30 min. Cell lysates were subjected to immunoblotting using antibodies against HIF-1α or actin. (**B**) Intensity of HIF-1α protein signals obtained in A was quantified using Eagle Sight densitometry software (Version 3.21; Stratagene). The HIF-1α densitometry data were normalized to those of the control (*Lane 1*) and actin levels. The plots represent the mean ± SD from three independent experiments. Calculation of HIF-1α half-life was performed by the regression program of Microsoft Excel 2000. (**C**) Cells were treated with 10–50 nM DT in the presence or absence of 1 mM MG132 in 20% and 1% O_2_ for 8 h. Cell lysates containing equal amounts of protein (20 mg) were separated by SDS-PAGE and immunoblotted with an anti-HIF-1α antibody. Actin was used as a loading control.

DT clearly inhibits accumulation of HIF-1a during hypoxia. To address a remaining question on how DT mediates such effect, we investigated its mechanism. X01 GSC were exposed to hypoxia for 8 h and subsequently treated with 100 mM cycloheximide (CHX), a protein synthesis inhibitor, under hypoxic conditions (Figure 3A). Hypoxia-induced accumulation of HIF-1a was rapidly reduced by treatment with CHX. In addition, combined CHX and DT, in comparison to CHX alone or DT alone, effectively decreased the intracellular levels of HIF-1a, even under hypoxic conditions (Figure 3B). Under normoxic conditions, HIF-1α is hydroxylated at Pro-402 and Pro-564 residues and is degraded rapidly by ubiquitination and subsequent association with the proteasome system [23, 24]. To investigate if inhibition of HIF-1α accumulation by DT under hypoxic conditions is mediated by the proteasome system, we used the proteasomal inhibitor MG132. Treatment with MG132 led to a significant increase of HIF-1α protein level in normoxic and hypoxic conditions (Figure 3C). DT inhibited MG132-mediated HIF-1α accumulation in a concentration-dependent manner in GSC. These results indicate that DT-induced HIF-1α depletion is not mediated by attened degradation of HIF-1α via the proteasome system. These data suggest that DT-induced inhibition of HIF-1a accumulation during hypoxia is not mediated by alteration of HIF-1a degradation, but rather by inhibition of protein synthesis.

### DT inhibits GSC angiogenic and invasive capacities

**Figure 4:**
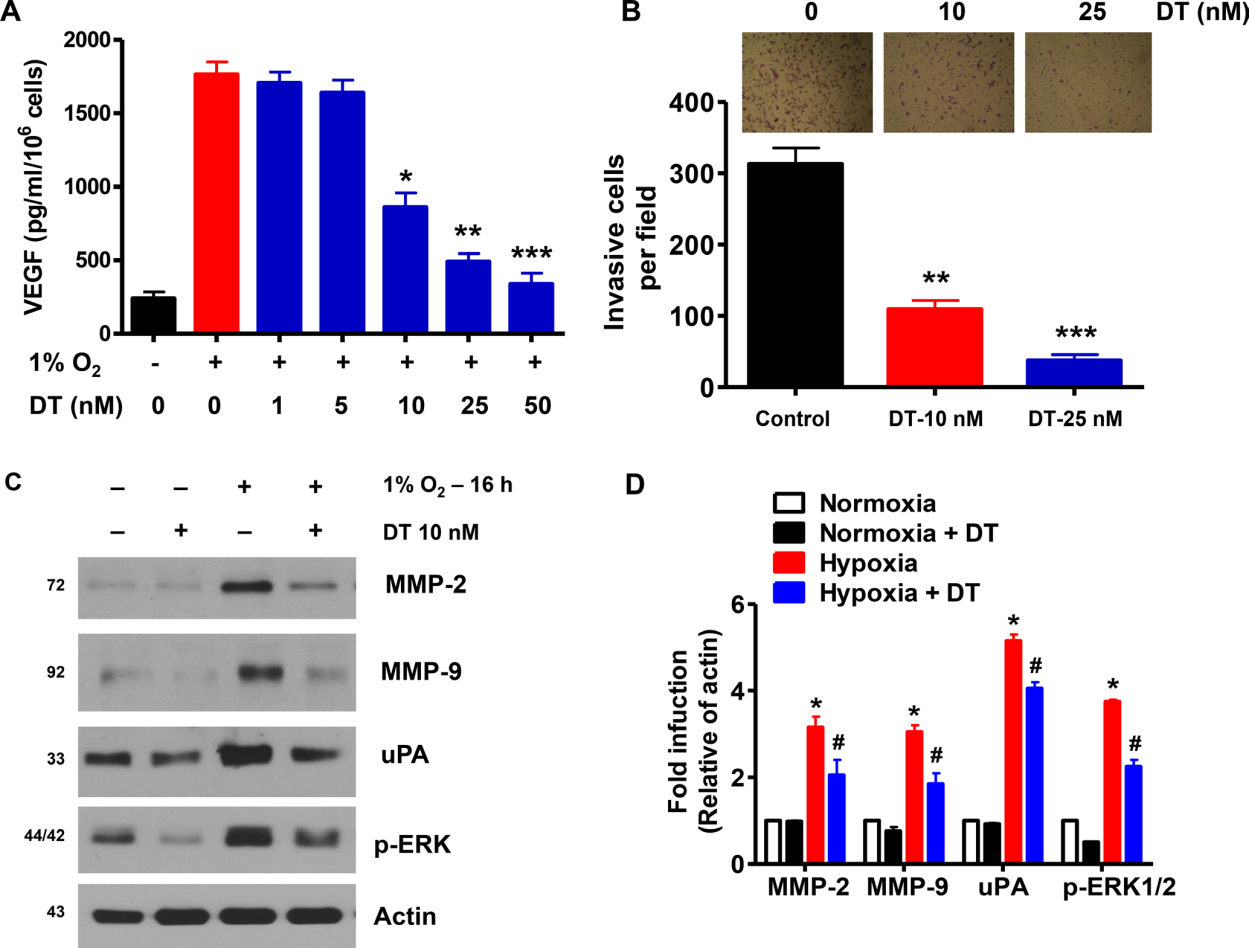
DT inhibits GSC angiogenic and invasive capacities (**A**) GSC were treated with various concentrations of DT (1–50 nM) for 16 h during hypoxia. Concentration of VEGF protein in the culture medium was determined by ELISA. The assays were performed in triplicates. (**B**) GSC were treated with DMSO or DT, and were cultured in sphere forming conditions in the upper well of matrigel-precoated transwell chambers for 16 h. Cells invading the lower well were fixed and stained with a Diff-Quick kit and photographed (100× magnification). Invasiveness was determined by counting cells in five randomly selected microscopic fields per well. (**C** and **D**) Western blot analysis shows that DT downregulates expression of invasion-related proteins in GSC during hypoxic conditions. Error Bars represent mean ± SD of triplicate samples. *P < 0.05, **P < 0.01, and ***P < 0.005.

VEGF, an immediate downstream target gene of HIF-1α playing a critical role in tumor angiogenesis [25], was increased in cell lysates and cell culture supernatants of GSC cultured under hypoxic conditions (Supplementary Figure 2A and 2B). Treatment of GSC with DT resulted in a dose-dependent decrease in hypoxia-induced VEGF expression at the protein level (Figure 4A). Such effect was confirmed in six additional cancer cell lines (Supplementary Figure 5A). We next examined effect of DT on hypoxia-induced cellular invasive and migratory activities [26, 27]. As shown in Figure 4B, an increase in the baseline invasiveness of GSC was observed under hypoxia However, pretreatment with DT suppressed hypoxia-stimulated invasiveness of GSC Figure 4C. In addition, we confirmed that DT suppresses expression of invasion-related proteins, MMP-2, MMP-9, uPA, and p-ERK, that are overexpressed in hypoxia (Figure 4C, 4D, Supplementary Figure 5B).

### DT inhibits GSC self-renewal capacity

**Figure 5:**
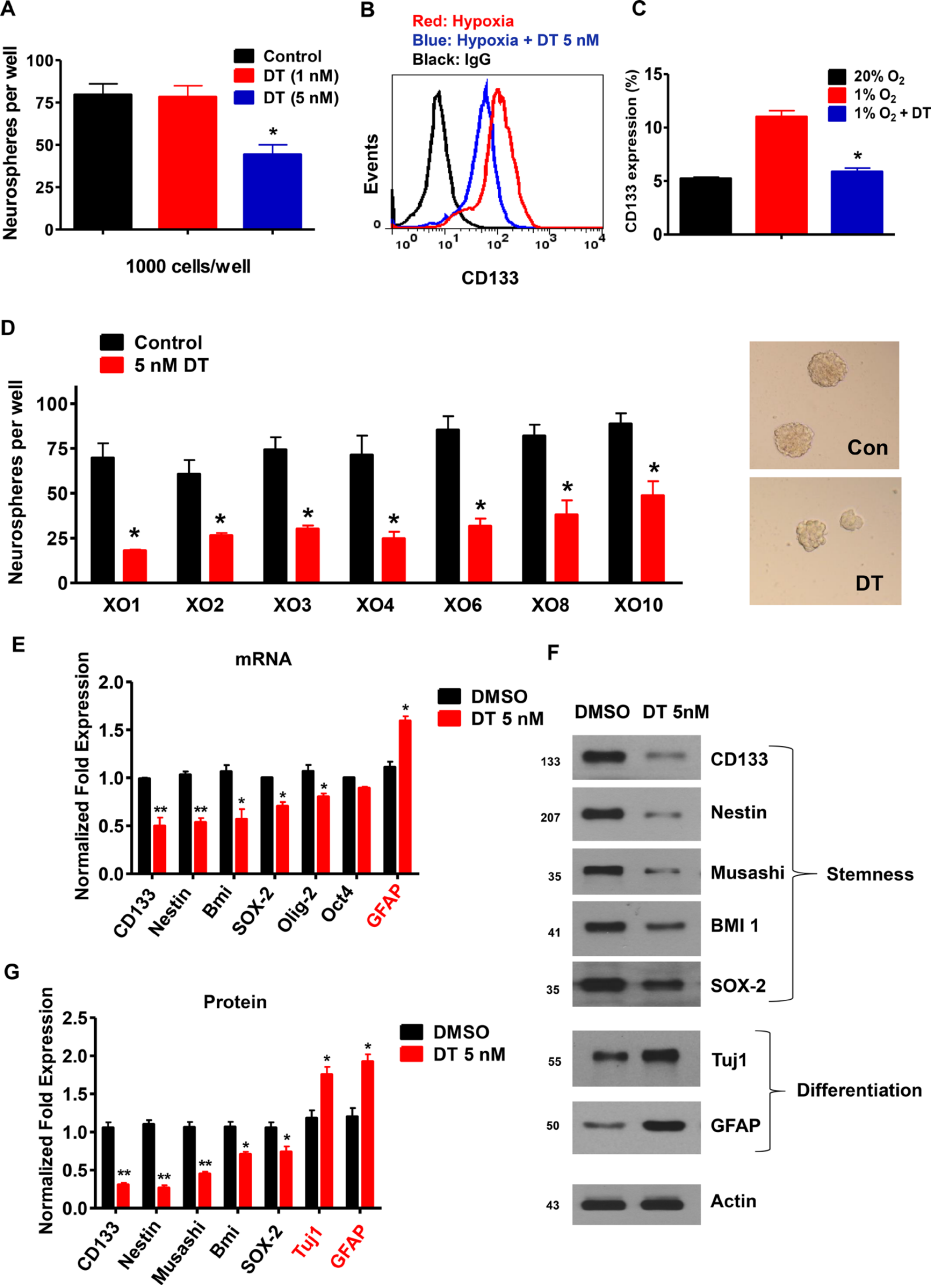
DT inhibits GSC self-renewal capacity (**A**) After 5 days of treatment with DT, neurospheres were quantified in 12 wells. Significant reduction in neurospheres were observed, relative to DMSO controls. Three independent experiments were performed. (**B** and **C**) GSC were treated with DMSO (control) or DT for 48 h. Flow cytometry demonstrated that DT was capable of abrogating hypoxic-induced expansion of CD133 positive cells. Each measurement was repeated in triplicates with three independent experiments. (**D**) Sphere-forming capacity, an *in vitro* surrogate of GSC self-renewal activity was strikingly reduced in various GSC upon incubation with DT. (**E**) mRNA levels of CD133, Nestin, Bmi-1, Sox-2, Olig-2, Oct4, and GFAP in GSCs at 48 h after DMSO or DT treatment. (**F** and **G**) Western blot for Nestin, CD133, Musashi-1, Bmi-1, Sox-2, Tuj1, and GFAP in GSCs at 48 h after DMSO or DT treatment. Actin was used as a loading control.

CD133 is a marker of GSC [28]. To evaluate the effect of DT on self-renewal capacity and survival of GSC, we analyzed sphere forming ability and apoptotic rate of CD133^+^ cells. Treatment with DT resulted in dramatic reduction in the CD133^+^ cell population (Supplementary Figure 3A). Although cell death was not significantly triggered by DT concentrations of 1–5 nM, there was an increase of cell death at a concentration of 10 nM (Supplementary Figure 3B). Thus, we used 5 nM of DT, a dose that does not cause cell death, for all subsequent experiments. Treatment of GSC with 5 nM DT caused significant reduction in the formation of spheres (Figure 5A) and the CD133^+^ cell population (Figure 5B and 5C). We also confirmed this in a broad representation of GSC (Figure 5D). Additional experiments designed to investigate expression of stem (CD133, Nestin, Bmi-1, SOX-2, Olig-2, and Oct4) and differentiation markers (GFAP and Tuj1) corroborated above results. We observed decrease in mRNA and protein expressions of stem markers and increase in differentiation markers upon exposure to DT (Figure 5E–5G).

### DT enhances the sensitivity of GSC to anticancer treatments

**Figure 6:**
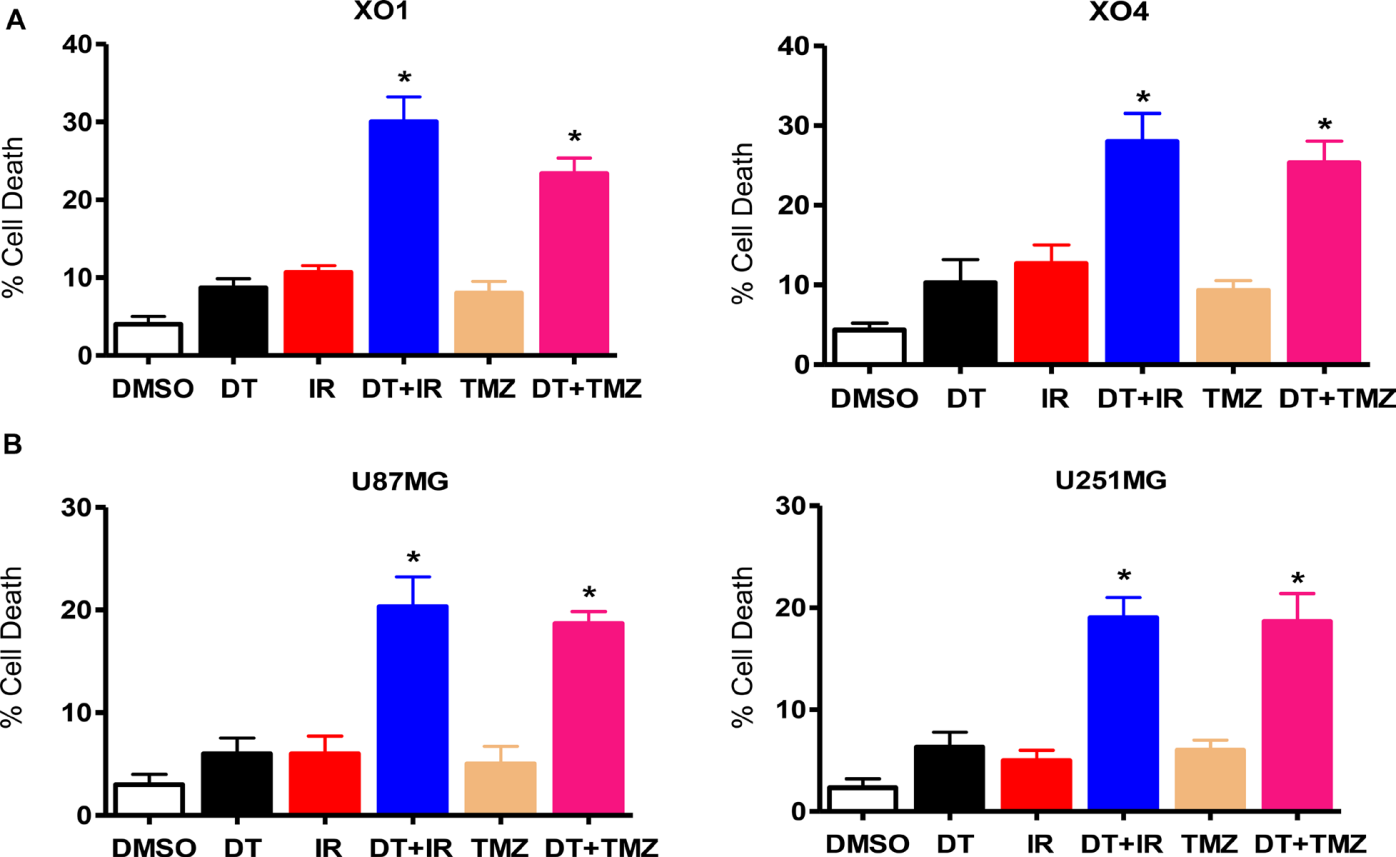
DT enhances the sensitivity of glioma cells to anticancer treatments Cell death was measured in two GSC lines (**A**) and two conventional glioma cell lines (**B**) by flow cytometry after propidium iodide staining. The percentage of propidium iodide positive cells increased when treated with DT before ionizing radiation (4 Gy) or TMZ (100 μM), in comparison to treatment with only DT, TMZ, or ionizing radiation. Error bars represent the mean ± SD of triplicate samples. *P < 0.01. TMZ: Temozolomide.

Since cancer stem-like cells are relatively resistant to chemotherapy and radiation therapy [29–32], we hypothesized that the suppression of stemness caused through DT treatment might sensitize GSC to ionizing radiation (IR) and Temozolomide (TMZ). To investigate the effect of DT on resistance to anticancer treatments, we treated GSC and glioma cells with DT in combination with IR or TMZ, which represent standard therapies for GB. As shown in Figure 6, treatment with DT alone did not cause significant cell death in GSC and glioma cells. However, DT in combination with IR or TMZ demonstrated striking enhancement of cell death.

### DT treated GSC have reduced capacity for tumor formation and attenuates HIF signaling in an orthotopic model

**Figure 7:**
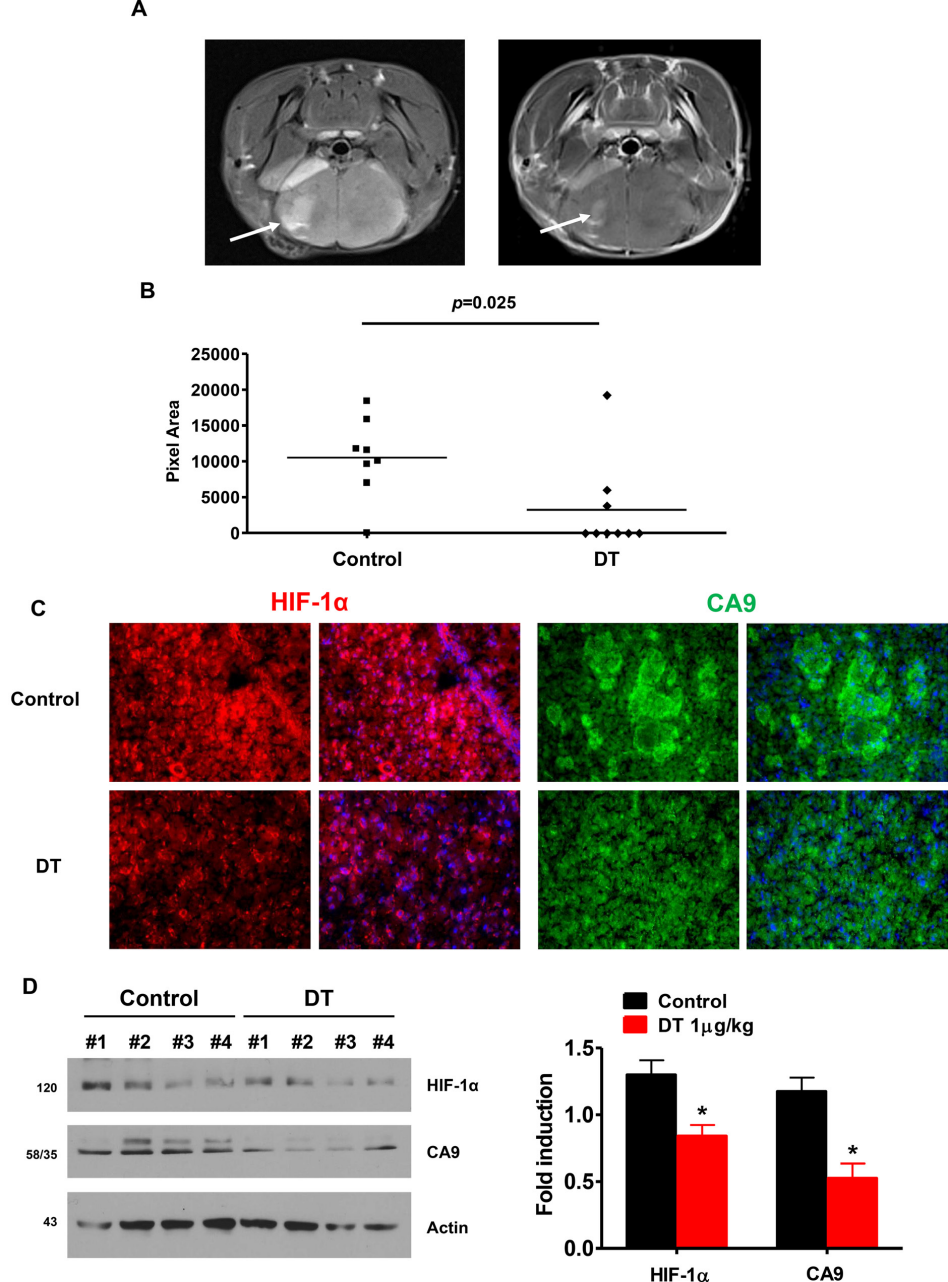
DT treated GSC have reduced capacity for tumor formation and attenuates HIF signaling in an orthotopic model (**A**) Tumor formation was monitored by MRI Arrow points to tumor. (**B**) Quantification of MR imaged tumor size. DT significantly reduced tumor size (*p* = 0.025). (**C**) HIF-1α and CA9 immunostaining in brain sections from vehicle- and DT-treated mice. (**D**) Anti-HIF-1α and anti-CA9 expression was assessed in four different protein samples using western-blotting (right), and the average value is shown on left. All experiments with statistical analyses were performed at least three times, and error bars depict means ± SD; *P < 0.05.

We assessed the therapeutic efficacy of DT in an orthotopic *in vivo* model using immunodeficient mice. Cells were implanted in the striatum of SCID mice (*n* = 8) and tumor sizes were measured after 4 weeks. We found that DT treated tumors were much smaller than vehicle treated control tumors. The tumor volume after 4 weeks reached ~1500 mm^3^ in control tumors, whereas in animals treated with DT at 1 μg/kg, the average tumor volume was 610 ± 56 mm^3^ (Figure 7A and 7B). As shown in Figure 7C and 7D, HIF-1α and CA9 expression was attenuated in the tumor exposed to DT.

## DISCUSSION

Intratumoral hypoxia is a critical therapeutic barrier. Low oxygen environment renders conventional therapies less effective and alters tumor cell metabolism in favor of acquiring a more aggressive phenotype. One mechanism by which hypoxia exerts poor clinical outcome in cancers is through enhanced survival of the cancer stem cell subpopulation. The hypoxic microenvironment promotes maintenance of GSC in a HIF-dependent mechanism [33]. Although the outcome from strategies to enhance intratumoral oxygenation have largely been disappointing, because the hypoxic adaptive cellular response is mediated by the hypoxia inducible factors, inhibiting the downstream signaling of these transcription factors may be worthwhile to explore. Since the cellular reaction to hypoxia and enhanced maintenance of GSC both converge at HIF signaling, we hypothesized that targeting this signaling network may disrupt GSC survival in low oxygen conditions. Here we report on the feasibility of inhibiting HIF-1α expression and its downstream signaling with a cardiac glycoside, digitoxin, in glioma stem cells.

The principal observations of this report are that HIF-1α expression and its downstream signaling is disrupted by digitoxin, and that exposure to digitoxin interferes with growth and function of GSC. In particular, digitoxin at clinically achievable concentrations interfered with HIF-1α accumulation in GSC subjected to hypoxia by preventing its protein synthesis. In addition, digitoxin downregulated hypoxia-induced activation of the extracellular signal-regulated kinase signaling cascade, a network with known crosstalk with hypoxia response. Also, the angiogenic and invasive capacities of GSC were attenuated by digitoxin. Exposure to digitoxin interfered with GSC self-renewal and enhanced sensitivity to both radiation and chemotherapy, two main modalities of GBM therapy. Lastly, these *in vitro* observations were validated in an orthotopic glioblastoma animal model.

Cardiac glycosides have long been appreciated for anticancer activities [34]. Proposed mechanisms of action are broad, and may vary depending on the cancer cell type and environmental context. These include alteration of K^+^, Na^+^, Ca^2+^ homeostasis, inhibition of glycolysis, increased production of reactive oxygen species, inhibition of topoisomerase II, upregulation of death receptors, alteration in membrane fluidity, increased level of p21, inhibition of NF-κB signaling, inhibition of Akt activation, down regulation of interleukin 8 receptor, induction of tumor cell differentiation, inhibition of soluble fms-like tyrosine kinase, and loss of mitochondrial membrane potential [13, 34, 35]. Series of unbiased library screening of compounds in search of novel anticancer agents have also identified cardiac glycosides as a promising family of agents [36]. Our previous effort to identify effective medical therapy for chordoma also unexpectedly identified cardiac glycosides [15]. Intriguingly, epidemiologic studies have uncovered evidence for cardiac glycosides in cancer prevention further validating its potential anticancer activity [12, 13, 37].

We focused on digitoxin in our glioblastoma studies because: 1) Unlike digoxin, digitoxin at a concentration achievable in human use was capable of inhibiting HIF-1α; 2) Digitoxin is a lipid soluble compound and likely able to penetrate the blood-brain-barrier. Glioblastoma is an incurable cancer with limited treatment options. Main reasons cited for its recalcitrant nature consists of intratumoral hypoxia, presence of resistant GSC, and poor delivery of therapeutics across the blood-brain-barrier. Digitoxin has the potential to address these therapeutic barriers, and as such may be deserving of further investigation as a therapeutic agent for glioblastoma.

## MATERIALS AND METHODS

### Reagents and antibodies

DT and TMZ were obtained from Sigma Chemical Co. (St. Louis, MO, USA). The proteasome inhibitor MG132 was purchased from Calbiochem (San Diego, CA, USA). Monoclonal antibodies were purchased from the following companies: anti-CA9, anti-PARP-1, and anti-uPA antibodies from Santa Cruz Biotechnology (Santa Cruz, CA, USA); anti-HIF-1α and anti-HIF-1β antibodies from BD Biosciences (San Jose, CA, USA); anti-phospho-JNK and anti-JNK antibodies from Promega (Madison, WI, USA); anti-actin antibody from ICN (Costa Mesa, CA, USA); anti-CD133 and anti-VEGF antibodies from Abcam (USA); anti-β-Tubulin (Tuj1) antibody from Covance (USA); anti-Nestin antibody from Chemicon (USA); anti-SOX-2 antibody from R&D Systems (Minneapolis, MN, USA); and anti-Bmi1, anti-Musashi, anti-GFAP, anti-phospho-ERK, anti-ERK, anti-phospho-p38, anti-p38, anti-MMP-2, anti-MMP-9, and anti-phospho-Akt antibodies from Cell Signaling (Beverly, MA, USA).

### Cell culture and hypoxic condition

Human GSC were established from acutely resected human tumor tissues [38, 39]. Tumor-sphere cultures were performed as described previously, with some modifications, in Dulbecco's modified Eagle's medium-F12 (Gibco-Invitrogen, La Jolla, CA, USA) containing penicillin G, streptomycin sulfate, B-27 (Gibco-Invitrogen), and recombinant human EGF and FGF (20 ng/ml; R&D Systems, Minneapolis, MN, USA). Cells were cultured at 37 °C, with 95% relative humidity, and 5% CO_2_. For hypoxic condition, 60 mm dishes containing cells were incubated in a hypoxic chamber (Forma Scientific, Marietta, OH) with a 94:5:1 mixture of N_2_/CO_2_/O_2_. Alternatively, cells were treated with 200 μM of the hypoxia mimetic, CoCl_2_, Sigma and DFO, Sigma.

### Drug treatments

Growing cells (70–80% confluence) in complete medium were treated with different concentrations of DT, followed by incubation in normal culture conditions or exposure to hypoxia (1% O_2_) for indicated time intervals according to the purpose of the experiment. To investigate the role of protein synthesis in suppression of hypoxia-induced HIF-1α in presence of DT, human GSC were exposed to hypoxic conditions for 8 h. This was followed by treatment with 100 μM of cycloheximide (CHX, Sigma) to suppress protein synthesis, in the presence or absence of 25 nM of DT for different time periods. To study the effect of DT on degradation of hypoxia-induced HIF-1α protein level, cells were pre-treated with 1 μM of MG132 for 30 min and cultured in presence of varying concentrations of DT for 8 h under normoxic and hypoxic conditions.

### Irradiation

Cells were plated in 60 mm dishes in culture medium and incubated at 37 °C with humidified conditions and 5% CO_2_. Cells were exposed to γ-rays at a final dose of 4 Gy using a ^137^Cs γ-ray source (*J.L. Shepherd & Associates (Model: MK1)*, San Fernando, CA, USA) at a dose rate of 2.27 Gy min^− 1^.

### Determination of cell viability

One or two days prior, cells were plated in triplicate in 60 mm dishes at 1 × 10^5^ cells/plate in 5 ml of tissue culture medium. For trypan blue exclusion assays, trypsinized cells were pelleted and resuspended in 0.2 ml of medium, 0.5 ml of 0.4% trypan blue solution, and 0.3 ml of phosphate-buffered saline solution (PBS). Samples were mixed thoroughly, incubated at room temperature for 5 min, and examined under a light microscope. At least 300 cells were counted for determination of survival.

### Cell viability assays

Cell viability was measured by flow cytometry using propidium iodide staining. All experiments were performed in triplicate.

### Knockdown and overexpression of ERK

ERK1/2 siRNA (Cat. No. SC-35335) and negative control siRNA (Cat. No. SC-37007) were obtained from SantaCruz Biotechnology (Santa Cruz, CA, USA). The human pcDNA 3.1-ERK was purchased from Addgene. Cells were transfected with siRNA oligonucleotides using Lipofectamine RNAi Max reagents (Invitrogen) according to the manufacturer's introductions. The XO4 cells were transfected in a stable manner with the pcDNA 3.1-ERK plasmid or the control plasmid pcDNA 3.1 using Lipofectamine as prescribed by the manufacturer (Invitrogen). After 24 h cells were treated with DT for further analysis.

### Transient transfection and luciferase assay

Cells were transiently transfected with a luciferase reporter plasmid pGL2-HRE [40] and a pRL-CMV reference *Renilla* luciferase plasmid (Promega, Madison, WI, USA) using Fugene HD (Roche Diagnostics), according to manufacturers’ instructions.

### VEGF ELISA

Human GSC were plated in 60 mm plates at a density of 2 × 10^5^ cells/ml in DMEM/F12 medium and incubated overnight before subjecting the cells to treatment. After treatment, the cell culture medium was removed for storage at -80 °C. Levels of VEGF protein in the medium were determined by ELISA using a commercial kit (R&D Systems, Minneapolis, MN, USA) according to manufacturer's instructions and our previous report [41]. All experiments were performed in triplicate.

### Proteome profiler array and immunoblot analysis

For Western blot analyses, we followed previously described protocol [42]. Proteins were separated by SDS-PAGE and electrophoretically transferred to a polyvinylidene fluoride (PVDF) membrane. The PVDF membrane was blocked with 5% nonfat dry milk in PBS-Tween-20 (0.1%, v/v) for 1 h. The membrane was incubated with primary antibody (diluted according to the manufacturer's instructions) at 4 °C overnight. Horseradish peroxidase-conjugated anti-rabbit or anti-mouse IgG was used as the secondary antibody. Immunoreactive protein was visualized by chemiluminescence (ECL, Amersham, Arlington Heights, IL, USA). The effect of DT on GSC was further assessed by the phosphorylation proteome profiler kit (R&D Systems) in accordance with the instructions of the manufacturer. Images were analyzed using ImageJ software (National Institutes of Health) and by subtracting PBS background levels (negative control) from sample signal levels. Experiments were performed in at least duplicate.

### Immunofluorescent staining

Immunohistochemistry of tumor tissue from mice was performed as previously described [1]. Antibodies used were human anti-HIF-1α (rabbit polyclonal antibody, 1:200) and anti-CA9 (rabbit polyclonal antibody, 1:200) from Novus Biologicals (Littleton, CO, USA) for the detection of hypoxia-induced proteins. Visualization was performed after incubation with Alexa fluorophore-conjugated secondary antibodies (1:1,000; Molecular Probes, Inc., Eugene, OR). Tissue slides were simultaneously stained with DAPI for identification of nuclei.

### Quantitative real-time PCR

Total cellular RNA was extracted using RNeasy Mini kit (Qiagen, Inc., Chatsworth, CA, USA) and purified using RNeasy columns according to the manufacturer's instructions. The integrity of RNA was assessed by agarose gel electrophoresis. TaqMan primers from Life Technologies Applied Biosystems (Foster City, CA) were purchased and used to measure gene expression (PROM1 (CD133): Hs01009261_m1; NESTIN: Hs00707120_s1; BMI1: Hs00995536_m1; SOX2: Hs01053049_s1; OLIG2: Hs00300164_s1; OCT4/POU5F1: Hs00999632_gH; GFAP: Hs00909238_g1; GAPDH: Hs03929097_g1). Total RNA (100 ng) was reverse transcribed using Superscript III first-strand synthesis supermix (Life Technologies, San Diego, CA, USA) according to the manufacturer's instructions. RT-PCR was performed using gene-specific TaqMan probes on an Applied Biosystems StepOne Plus Real-Time PCR system with TaqMan PCR Master Mix (Life Technologies). The thermal cycling conditions were as follows: 95°C for 5 min, followed by 40 cycles of 95°C for 10 s, 55°C for 10 s and a final step of 72°C for 30 s. Samples were amplified in triplicate and data were analyzed using Applied Biosystems StepOne software V2.1 (Life Technologies). Ct values for all genes were normalized to those of HTERT from the same RNA sample. Average HTERT Ct values for each RNA sample were used to derive a scaling factor for normalization of the results derived from individual RNA samples.

### Invasion assays

Transwell invasion experiments were performed using 24-well BD BioCoat^®^ Growth Factor Reduced Matrigel^®^ Invasion Chambers (BD Biosciences, Bedford, MA, USA). The invasion chambers consisted of a BD Falcon^®^ cell culture insert with an 8-μm pore size PET membrane coated with BD Matrigel Matrix, which serves as a reconstituted basement membrane *in vitro*. Tumor cells, under normoxia or hypoxia, were prepared as cell suspensions in serum-free culture medium with laminin at a density of 1 × 10^5^ cells/ml with or without DT. Membranes, with cells attached to the lower surface (invaded cells), were fixed with methanol and stained with hematoxylin. Invasiveness was determined by counting cells in five microscopic fields per well, and the extent of invasion was expressed as an average number of cells per microscopic field. Cells were imaged with a phase-contrast microscope (Leica Microsystems, Bannockburn, IL, USA). All experiments were performed in triplicate.

### Sphere-forming assays

Self-renewal activity of human GSC in response to DT was assessed by sphere formation. For this assay, human GSC were seeded at 1000 cells per well in a 24-well plate in serum-free medium with or without DT and cultured for 5 days.

### FACS analysis for expression of CD133

Human GSC (1 × 10^6^ cells) were plated in appropriate medium in 60 mm plates in triplicate and were allowed to recover overnight. Human GSC were treated with a DMSO control or increasing concentrations of DT for 48 h. To further characterize the effect of DT on human GSC, each sample was labeled with phycoerythrin-conjugated anti-human CD133/1 (AC133) antibody (Miltenyi Biotec, Auburn, CA, USA) with phycoerythrin-secondary antibody (BD Biosciences, San Jose, CA, USA) according to the manufacturer's recommendation. Appropriate compensation and isotype controls were used. All experiments were performed in triplicate.

### *In vivo* tumor formation

Human GSC (3 × 10^5^) were stereotactically implanted into the striatum of immunodeficient mice (*n* = 8). One week post-tumor implantation, the animals were treated with i.p. injections of 1 μg DT or vehicle control once a day for 3 weeks. Magnetic resonance imaging study was performed after 3 weeks of treatments. Animals were sacrificed 4 weeks after tumor implantation. The brains were removed, sectioned, and visualized by immunostaining. Maximal tumor cross-sectional areas were measured by computer-assisted image analysis and tumor volumes were calculated.

### Statistical analysis

Statistical analyses were performed using GraphPad InStat 5 software (GraphPad Software, Inc., San Diego, CA, USA). Values reported as the mean ± SD. *P* < 0.05 was considered significant, and is indicated by asterisk in the figures.

## SUPPLEMENTARY MATERIALS FIGURES


