# Supplementary material for: Cardiac glycosides suppress the maintenance of stemness and malignancy via inhibiting HIF-1α in human glioma stem cells
Source: Oncotarget. 2017 Mar 30;8(25):40233–45. doi: 10.18632/oncotarget.16714 (PMC5522201; doi:10.18632/oncotarget.16714)
Supplement: Supplementary file 1 [file oncotarget-08-40233-s001.pdf]

Cardiac glycosides suppress the maintenance of stemness and malignancy via inhibiting HIF-1α in human glioma stem cells

Supplementary Materials

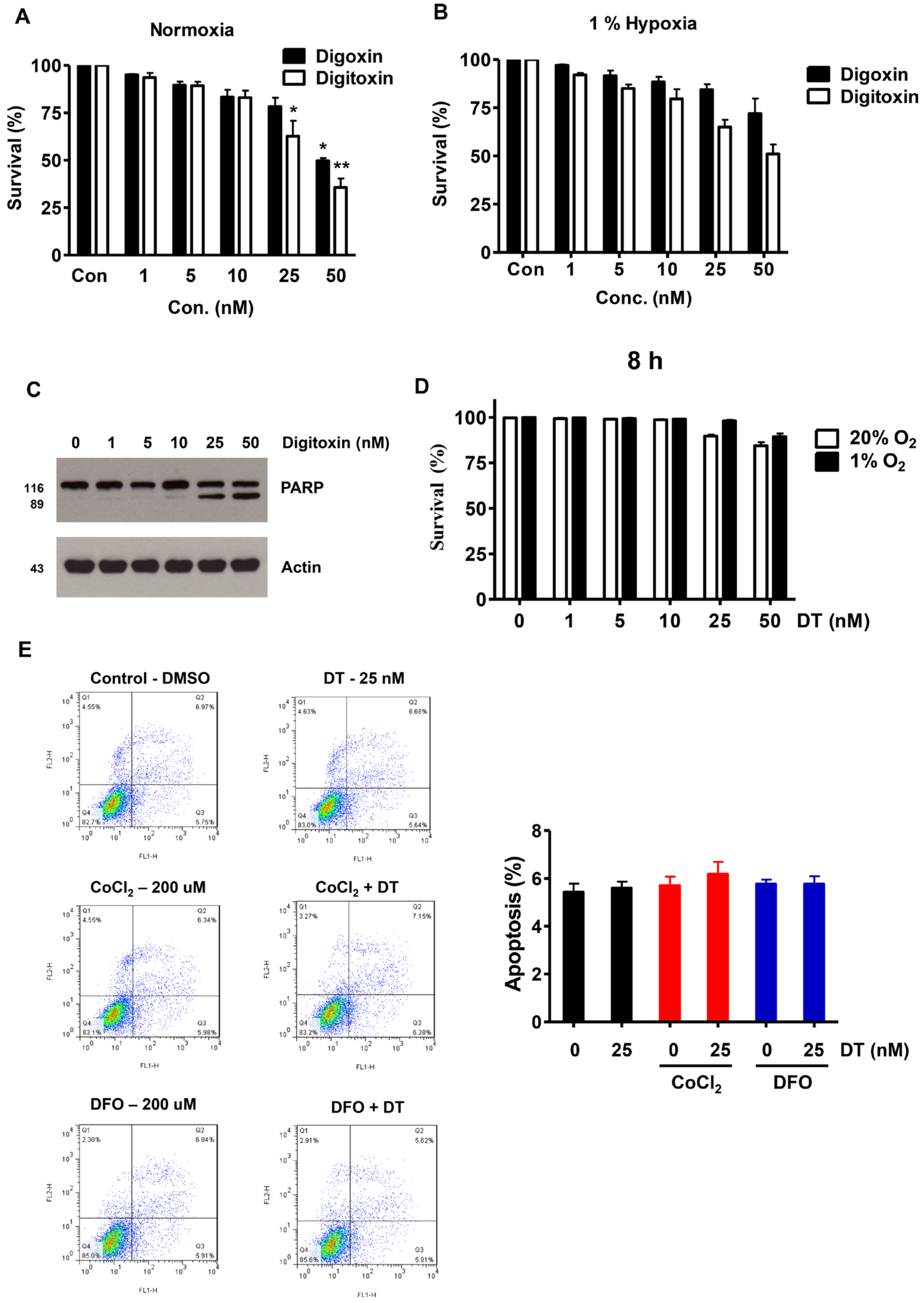

Supplementary Figure 1: Cardiac glycosides induce cytotoxicity in GSCs. Survival was analyzed by the trypan blue dye exclusion assay as described in materials and methods. Error bars represent the mean ± SE from three separate experiments.

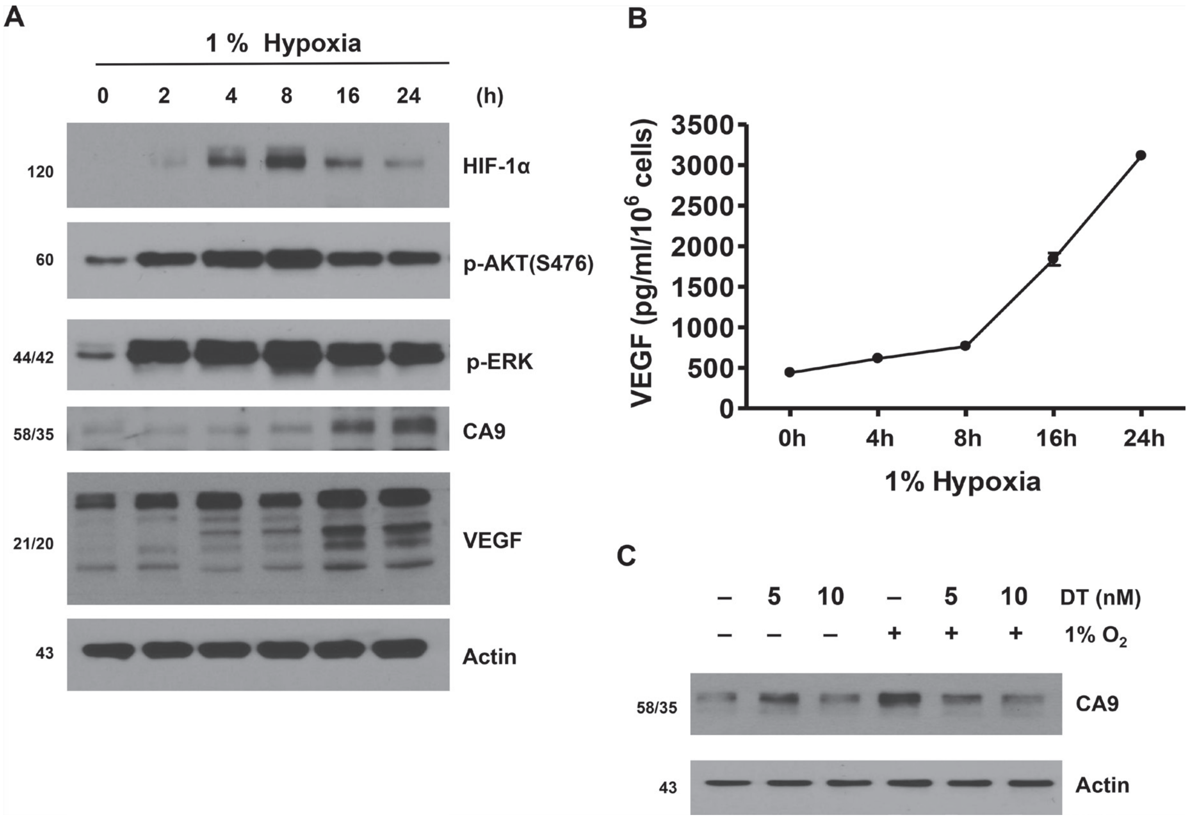

Supplementary Figure 2: Kinetics of HIF-1a accumulation and its downstream signaling in hypoxic conditions. Cells were exposed to 1% O_2 for various times (0–24 h). (**A**) Western blot for anti-HIF-1α, anti-phospho-AKT, anti-phospho-ERK, anti-CA9, and anti-VEGF in GSCs. Actin was used as a loading control. (**B**) Concentration of VEGF in the culture medium was determined by ELISA. Assays were performed in triplicates. (**C**) GSCs were treated with DT (5 and 10 nM) for 16 h during hypoxia. Western blot of GSC for anti-CA9 is shown. Actin was used as a loading control.

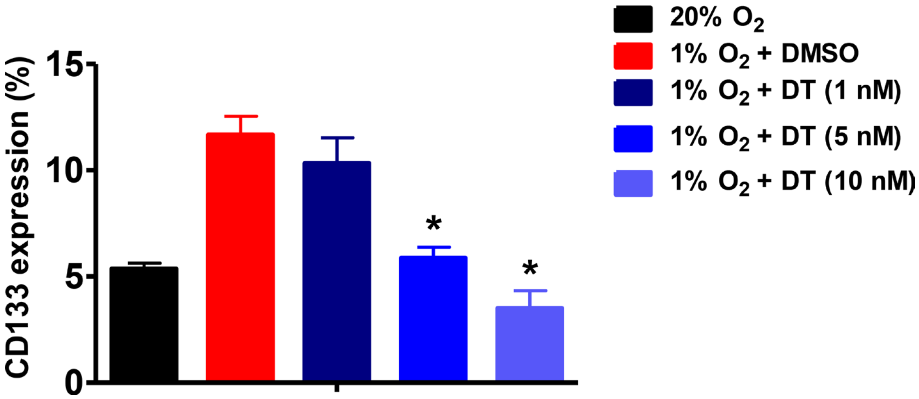

Supplementary Figure 3: DT abrogates hypoxia-induced CD133 cell expansion. Quantification of CD133+ cell population by flow cytometry shows that DT effectively suppresses CD133+ cell expansion induced by hypoxia.

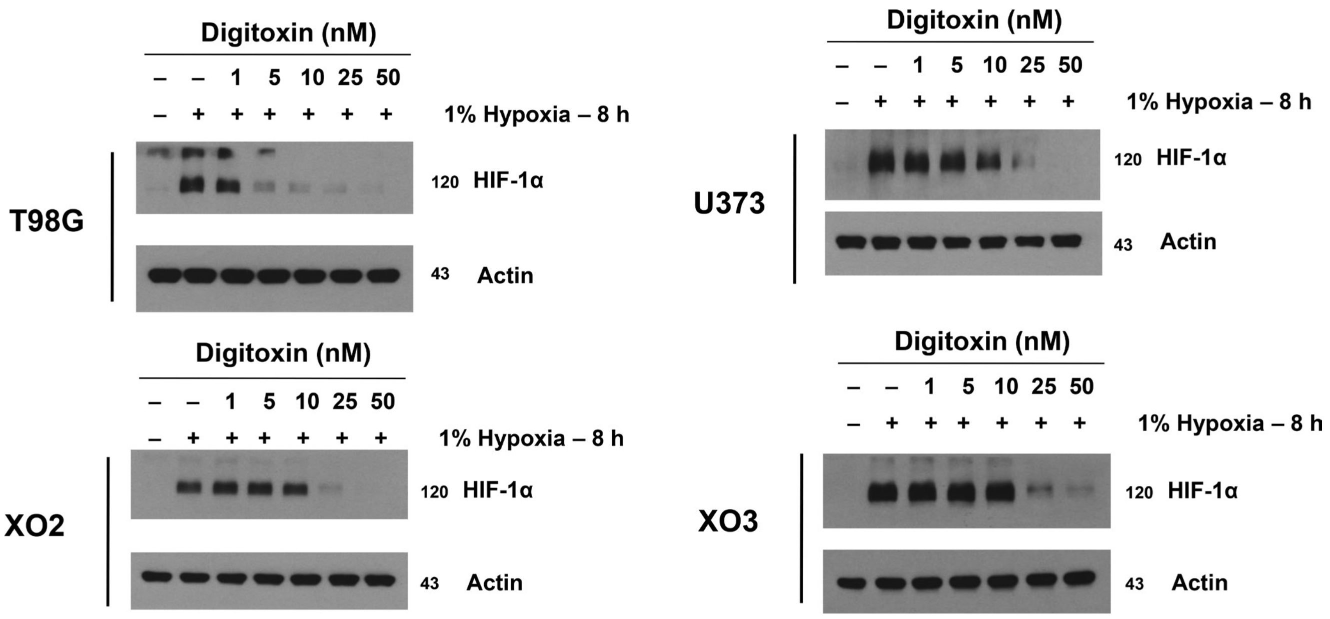

Supplementary Figure 4: DT inhibits HIF-1α accumulation during hypoxia in various glioma cell lines. T98G, U373, XO2, and XO3 cells were exposed to 1% O_2 and treated with 1–50 nM DT for 8 h. Cell lysates containing equal amounts of protein (20 mg) were separated by SDS-PAGE and immunoblotted with an anti-HIF-1α antibody. Actin was used as a loading control.

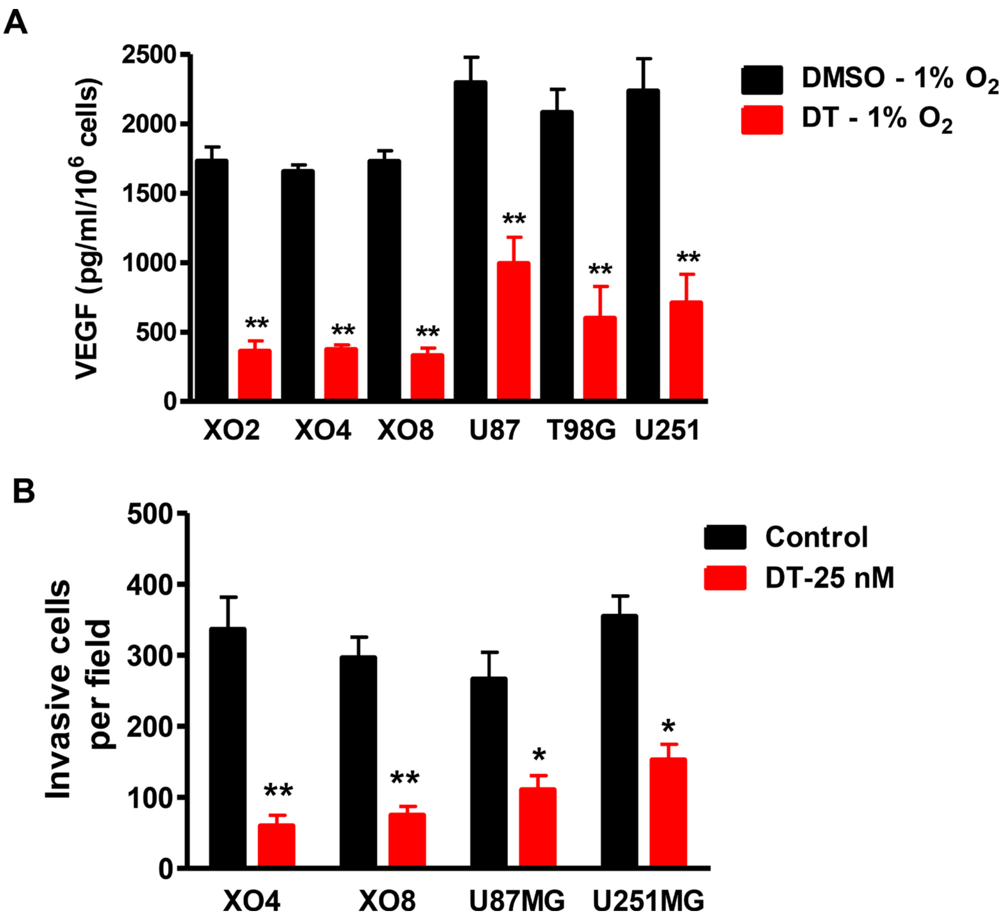

Supplementary Figure 5: DT broadly suppresses angiogenic and invasive capacities in cancer cell lines. (**A**) Cells were treated with DT (25 nM) for 16 h during hypoxia. The concentration of VEGF in the culture medium was determined by ELISA. Assays were performed in triplicates. (**B**) Cells were treated with DMSO or DT (25 nM), and were cultured in sphere forming conditions in the upper well of a matrigel-precoated transwell chamber for 16 h. Assays were performed in triplicates.

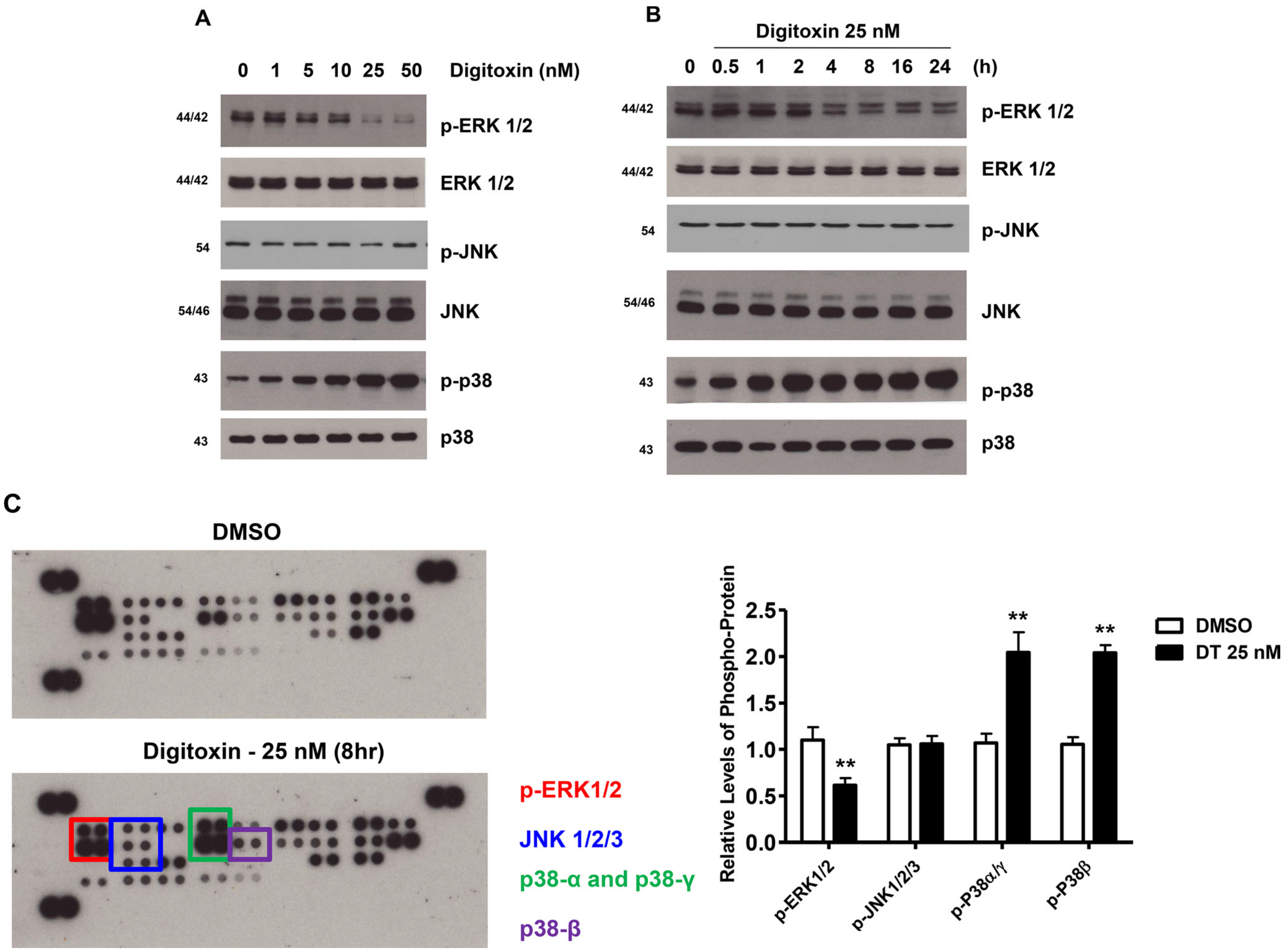

Supplementary Figure 6: Effect of DT on ERK signaling in GSC. (**A**) GSCs were treated with various concentrations (1–50 nM) of DT for 24 h. (**B**) GSC were treated with 25 nM DT for various time periods (0.5–24 h). Western blot analysis was performed using anti-phospho-ERK, anti-phospho-JNK, and anti-phospho-p38 antibodies. Lysates containing equal amounts of protein (20 μg) were separated by SDS-PAGE and immunoblotted. Actin was used to confirm the equal amount of proteins loaded in each lane. (**C**) Phospho-protein array: GSCs were treated with vehicle or 25 nM DT. Cells were lysed and 250 μg of protein was loaded onto the array. The phospho-protein levels were determined using the antibody cocktail provided by the manufacturer. The blots are representative of two separate experiments. The relative phospho-protein levels (phospho-protein/total protein) were determined by densitometry analysis. $^{**}P < 0.01$ versus DT-treated cells.
